# Supplementary material for: Integrated lipidomics and RNA sequencing analysis reveal novel changes during 3T3-L1 cell adipogenesis
Source: PeerJ. 2022 May 3;10:e13417. doi: 10.7717/peerj.13417 (PMC9074861; doi:10.7717/peerj.13417)
Supplement: Supplemental Information 1 [file peerj-10-13417-s001.pdf]

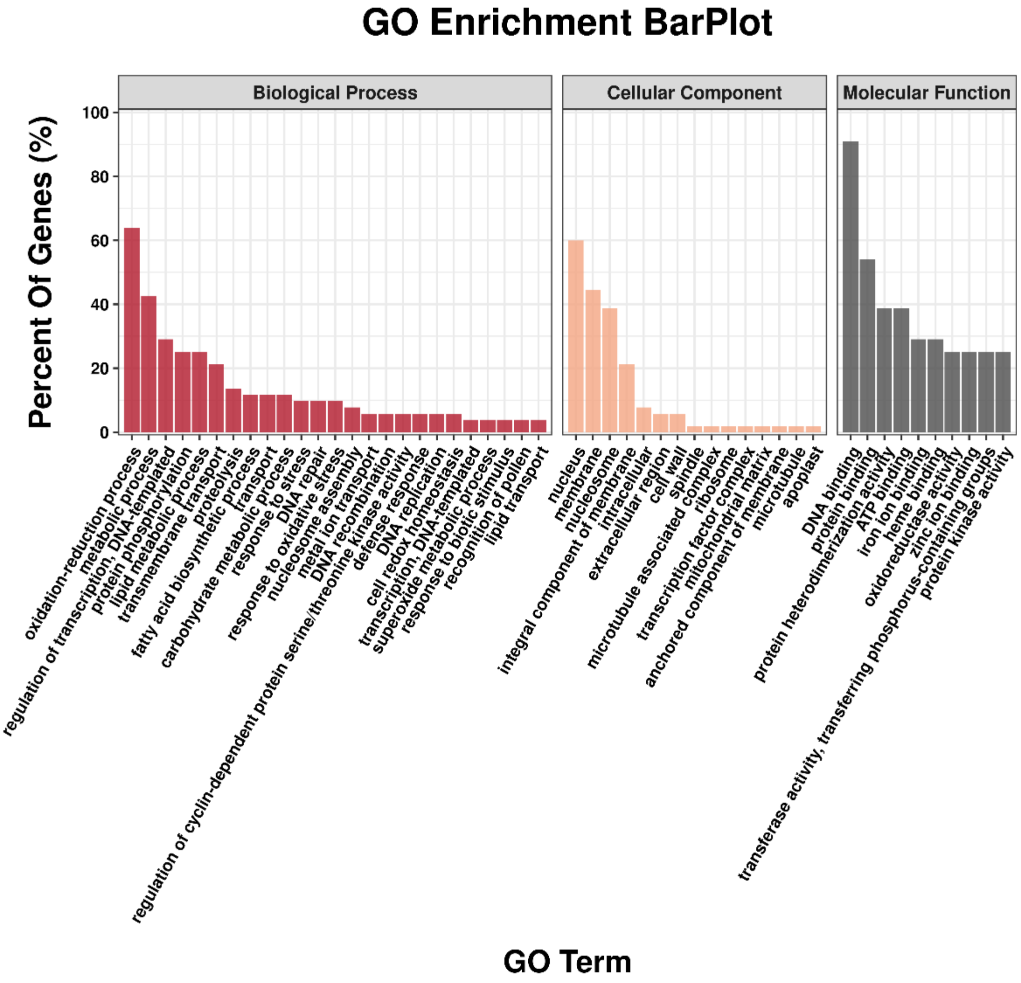

Figure S1 Gene ontology (GO) term enrichment analysis.

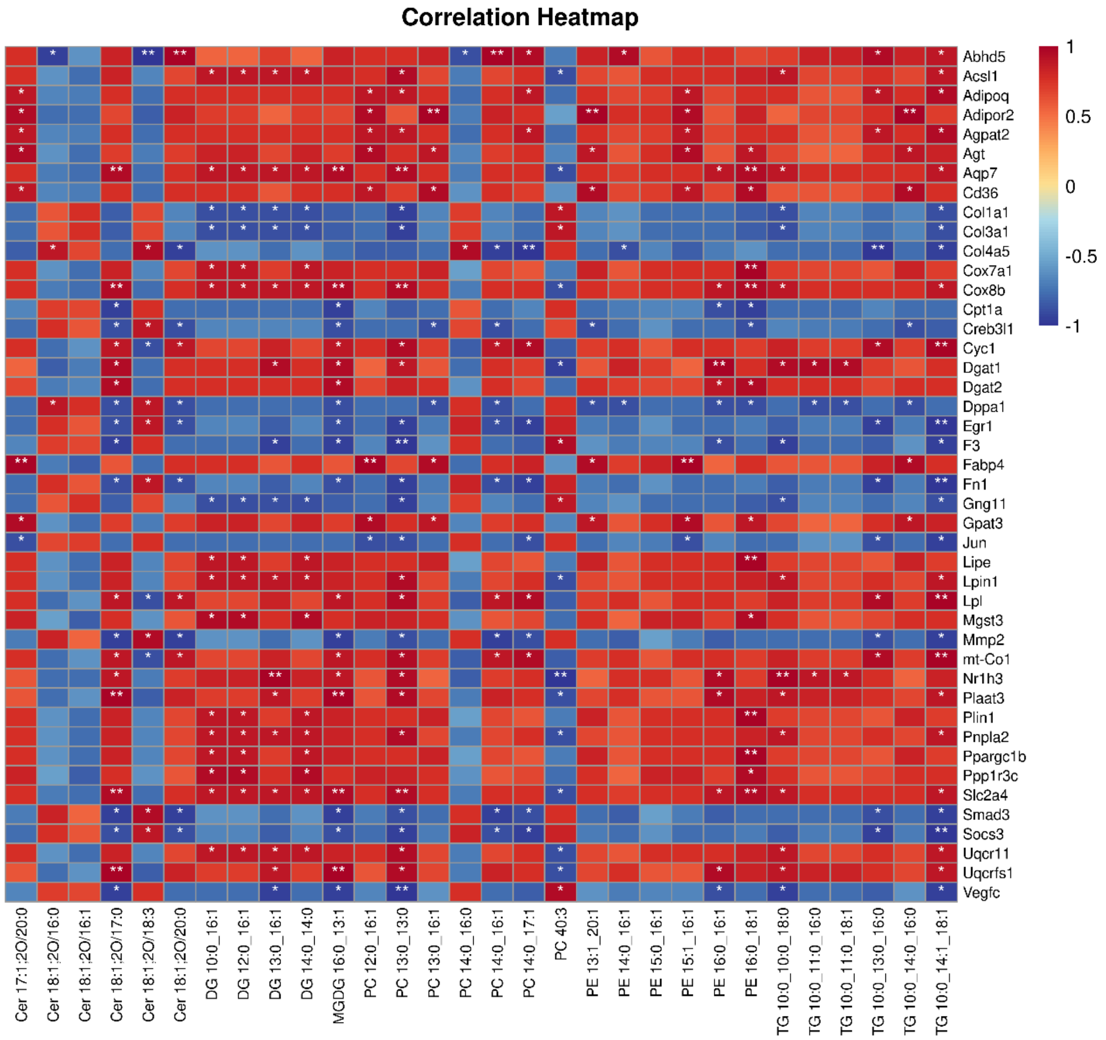

Figure S2 Heat map of correlation analysis between differentially expressed genes and lipid metabolites

Positive correlations are blue and negative correlations are red.*<0.05, **<0.01.

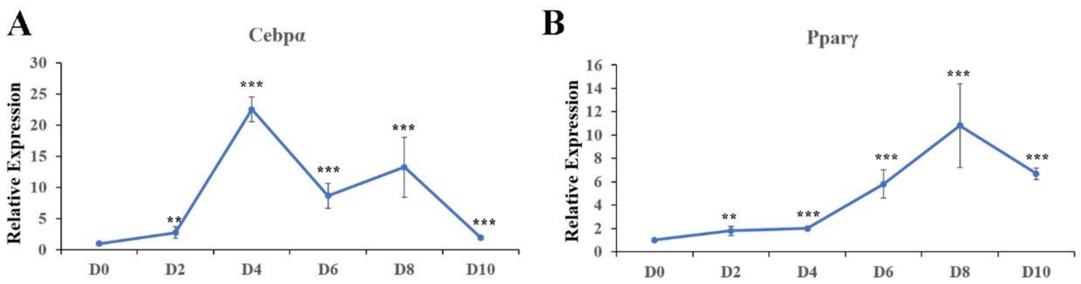

Figure S3 The expression of *Cebp/α* and *Pparγ* in adipogenesis detected by RT-qPCR
